# Retraction Note: Surface modification of thin film composite forward osmosis membrane using graphene nanosheets for water desalination

**DOI:** 10.1038/s41598-023-30298-4

**Published:** 2023-02-28

**Authors:** Fatma Mohamed El-Sayed, Mohamed E. A. Ali, Heba Isawi, M. M. Abo Aly, M. M. S. Abo El-Fadl

**Affiliations:** 1 Hydrogeochemistry Department, Desert Research Center, Cairo, Egypt; 2 Chemistry Department, Faculty of Science, Ain Shams University, Cairo, Egypt

Retraction of: *Scientific Reports* 10.1038/s41598-022-25700-6, published online 08 December 2022

The Editors have retracted this Article at the request of the authors.

After the publication of this Article it was brought to the Editors' attention that:

- the results shown in Figure 1 were taken from^1^ where they are described as representing a different sample;
- the data shown in Figure 3E are a duplication of an image in Figure 4E in^2^.

The Editors therefore no longer have confidence in the results reported in the Article.

Fatma Mohamed El-Sayed, Mohamed E.A.Ali, Heba Isawi, and M. M. Abo Aly agree with this retraction and its wording. M. M. S. Abo El-Fadl has not responded to correspondence from the Editors about this retraction.
